# A method for developing standardised interactive education for complex clinical guidelines

**DOI:** 10.1186/1472-6920-12-108

**Published:** 2012-11-06

**Authors:** Janet I Vaughan, Heather E Jeffery, Camille Raynes-Greenow, Adrienne Gordon, Jane Hirst, David A Hill, Susan Arbuckle

**Affiliations:** 1 Maternal-Fetal Medicine Unit, John Hunter Hospital, Lookout Road, New Lambton, NSW, 2305, Australia; 2 Sydney School Public Health, Edward Ford Building, University of Sydney, Sydney, NSW, 2006, Australia; 3 RPA Newborn Care, Royal Prince Alfred Hospital, Missenden Road, Camperdown, NSW, 2050, Australia; 4 Sydney Medical School, University of Sydney, Royal North Shore Hospital, Reserve Road, St Leonards, NSW, 2065, Australia; 5 Sydney Medical School, Edward Ford Building University of Sydney, Sydney, NSW, 2006, Australia; 6 Histopathology Department, The Children’s Hospital at Westmead, Cnr Hawkesbury Rd and Hainsworth St, Westmead Sydney, NSW, 2145, Australia

**Keywords:** Practice guidelines as a topic, Implementation, Information dissemination, Education medical continuing, Interprofessional education, Action research, Perinatal mortality, Stillbirth, Fetal death

## Abstract

**Background:**

Although systematic use of the Perinatal Society of Australia and New Zealand internationally endorsed Clinical Practice Guideline for Perinatal Mortality (PSANZ-CPG) improves health outcomes, implementation is inadequate. Its complexity is a feature known to be associated with non-compliance. Interactive education is effective as a guideline implementation strategy, but lacks an agreed definition. SCORPIO is an educational framework containing interactive and didactic teaching, but has not previously been used to implement guidelines. Our aim was to transform the PSANZ-CPG into an education workshop to develop quality standardised interactive education acceptable to participants for learning skills in collaborative interprofessional care.

**Methods:**

The workshop was developed using the construct of an educational framework (SCORPIO), the PSANZ-CPG, a transformation process and tutor training. After a pilot workshop with key target and stakeholder groups, modifications were made to this and subsequent workshops based on multisource written observations from interprofessional participants, tutors and an independent educator. This participatory action research process was used to monitor acceptability and educational standards. Standardised interactive education was defined as the attainment of content and teaching standards. Quantitative analysis of positive expressed as a percentage of total feedback was used to derive a total quality score.

**Results:**

Eight workshops were held with 181 participants and 15 different tutors. Five versions resulted from the action research methodology. Thematic analysis of multisource observations identified eight recurring education themes or quality domains used for standardisation. The two content domains were curriculum and alignment with the guideline and the six teaching domains; overload, timing, didacticism, relevance, reproducibility and participant engagement. Engagement was the most challenging theme to resolve. Tutors identified all themes for revision whilst participants identified a number of teaching but no content themes. From version 1 to 5, a significant increasing trend in total quality score was obtained; participants: 55%, p=0.0001; educator: 42%, p=0.0004; tutor peers: 57%, p=0.0001.

**Conclusions:**

Complex clinical guidelines can be developed into a workshop acceptable to interprofessional participants. Eight quality domains provide a framework to standardise interactive teaching for complex clinical guidelines. Tutor peer review is important for content validity. This methodology may be useful for other guideline implementation.

## Background

The global burden of stillbirth currently estimated at 2.64 million per year has gained recent international attention [1]. The magnitude in Australia has not changed over the past two decades [2] with many of the underlying causes unknown because a significant proportion of stillbirths are not investigated appropriately [3]. To assist clinicians in the investigation and audit of perinatal deaths the Perinatal Society of Australia and New Zealand developed the Clinical Practice Guideline for Perinatal Mortality (PSANZ-CPG) [4]. Its systematic use has been shown to reduce stillbirth classified as unexplained by two-thirds i.e. from 34 to 13% [5]. However, the PSANZ-CPG is a long (154 pages) and complex publication providing recommendations for clinicians working in maternity services including obstetricians, midwives, neonatologists, neonatal nurses, pathologists, paediatricians, general practitioners and social workers/bereavement counsellors [4]. Survey evidence from doctors and midwives shows inadequate implementation of PSANZ-CPG at the hospital level with only 42% of respondents even being aware of them [6]. Franke et al. have performed a systematic meta-review to understand factors which influence the implementation of guidelines. The major guideline characteristic associated with non-compliance is complexity with the challenge for developers being to produce usable and understandable guidelines when they are aimed at different target groups with varying educational levels and backgrounds [7].

Inadequate implementation of clinical guidelines is a well acknowledged problem but research into implementation strategies is limited [8-10]. With only 19% of those aware of the PSANZ- CPG having received training in its use, education was the preferred method stated by 90% of survey respondents for improving uptake [6]. Similarly a US survey of specialist obstetricians recommended implementing education strategies to improve care as only 30% of respondents were very comfortable in their knowledge of causes, prevention and management of stillbirth [11]. A recent systematic review of implementation strategies identified that interactive education was consistently effective at achieving changes in clinical processes but acknowledged the lack of clarity in “the most effective mix in interactive education” [12]. We have experience with a teaching method called SCORPIO (Structured, Clinical, Objective Referenced, Problem-oriented, Integrated and Organised) [13]. It combines a mix of interactive and didactic teaching embedded in a framework based on psychological evidence that maximises adult learning [14]. Its effectiveness in changing clinical behaviour to improve health outcomes in a interprofessional clinical setting has been demonstrated [15] but it has not previously been used as a methodology to implement clinical guidelines.

The first major challenge in developing this workshop was simplification of the complexity of content so implementation retained the capacity to improve the intended health outcomes. Secondly the education needed to be relevant to all of the interprofessional participants. Our aim was to transform the PSANZ-CPG into a SCORPIO based education workshop to develop quality standardised interactive education acceptable for learning skills in collaborative interprofessional care.

## Methods

The construct for our *Perinatal Loss Workshop* included an educational framework, guideline transformation process and tutor training methodology.

### Educational framework

SCORPIO is a method for teaching a defined curriculum through a series of stations in which trained tutors interact with participants to learn skills [13]. It is based on a module consisting of a study guide, teaching stations and formative assessment representing the three components required for adult learning [14]. As the original SCORPIO methodology was designed for medical student teaching, this study has used a modified methodology for postgraduate interprofessional learning [15].

#### Study guide

The study guide, containing the teaching aims and the learning objectives for each teaching station, is distributed to participants a number of days before the workshop activating their prior knowledge and informing them of the organizational structure of the workshop.

#### Teaching stations

The content is delivered using mixed didactic-interactive teaching and ideally each station incorporates at least one different interactive methodology to encourage sustained participant engagement. Examples include: using models for supervised physical examination, using clinical specimens for anatomical examination, structuring a role play to involve all participants, debating an issue, physically plotting measurements on graphs and discussion based on participant experiences. Groups rotate around all of the stations with a break halfway. Ideally each group has 6 participants, but can function adequately with 5. With 6 stations, 36 participants is the maximum number per workshop, but with rest stations and different station rotation formats a minimum of 15 can be accommodated. Each of the 6 teaching stations is structured to teach a specific skill integrated within the workshop topic. Psychomotor, cognitive or attitudinal skills can all be incorporated in the SCORPIO circuit [13]. Each of 6 content expert tutors teaches their allocated station using either the sequence of “tell, show, do, feed-back” or the hypothetico-deductive reasoning sequence embedded in problem-based learning [16]**.** Prior to the teaching stations the workshop commences with a 15 to 20 minute didactic lecture to the whole group providing an overview of the curriculum topic and informing them of the context and process for the subsequent teaching stations.

#### Formative assessment

After completing the teaching stations, a formative assessment with feedback is used as learning enhancement to ensure knowledge and understanding of the topic has been achieved [13]. We used structured short answers with immediate feedback in an interactive group session.

### Guideline transformation process

The PSANZ-CPG specifically addresses investigation, audit and psychosocial aspects of bereavement to enhance both the accuracy of information about the causes of perinatal death and the quality of care for parents and families [4]. The core information for transformation to six skill-based topics suitable for teaching using the SCORPIO methodology was extracted from key recommendations summarised in the PSANZ-CPG as sections [4]. Each topic formed the basis of a teaching station with one teaching aim and three learning objectives (HEJ, AG). The content within each teaching station was matched to the relevant PSANZ-CPG section and contextualised using clinical examples and/or scenarios. Focussing on essential skills maximised clinical relevance to interprofessional participants. All the content was integrated to form a curriculum that operates in a highly organised framework.

### Tutor training

Tutors were selected on the basis of their prior experience with SCORPIO teaching and/or their status as opinion leaders, including consumer group representation. Tutors were required to comply with the SCOPRIO methodology and respond effectively to feedback. Tutors engaged in a series of meetings for the purpose of being instructed in the SCORPIO methodology and practicing their teaching station to ensure that the whole workshop is coordinated and well-timed.

### Standardised interactive education

**Figure 1:**
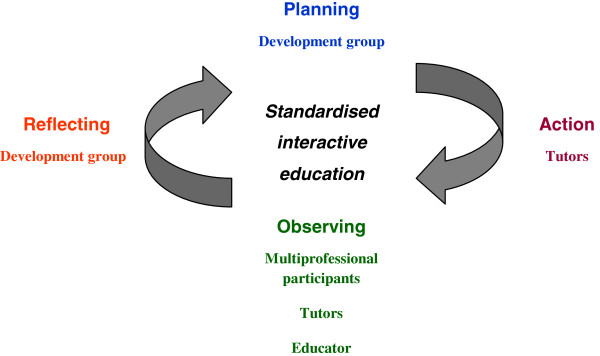
Participatory action research cycle.

Standardised interactive education we defined as the development of content and teaching standards. Content standards are the knowledge and skills itemised in the curriculum. Teaching standards are the processes which optimise delivery to participants. The core development group comprised 5 clinicians representative of the PSANZ-CPG target audience [JIV,HEJ,AG, JH, SA]. Using participatory action research through a recurring cycle of planning, acting, observing and reflecting [17,18] the ongoing role of the development group was to modify the *Perinatal Loss Workshop* construct to develop quality standardised interactive education (Figure 1). Contributions to the action research process were derived empirically and produced progressively different versions of the workshop.

#### Pilot workshop

After the initial PSANZ-CPG transformation and tutor training was completed a pilot workshop was held to clarify its relevance and acceptability to opinion leaders and policy makers. Key stakeholders were invited including the PSANZ-CPG authors [4], and representatives from the Commonwealth Department of Health and Aging, Royal Australian and New Zealand College of Obstetrics and Gynaecology (RANZCOG), Australia and New Zealand Stillbirth Alliance (ANZSA), Stillbirth Foundation and other consumer groups. Formal evaluation from these interprofessional, participant comments after this workshop provided collaborative critical observation for reflection by the development group (Figure 1).

#### Subsequent development workshops

Further workshops were held in response to requests from ANZSA and RANZCOG on an ad hoc basis. These workshops provided the opportunity for an empirical mixed methods approach to evolve with feedback from three sources being: interprofessional participants, tutor peers and an independent educator (DH) (Figure 1). Feedback from each source included formalised written responses to the three criteria of “best aspects”, “worst aspects” and “suggestions for improvement” of the workshop. Responses were collated in a written report and distributed to the development team, who used the information to reflect critically on the *Perinatal Loss Workshop* construct and direct planning and acting in the action research cycle revision process (Figure 1). For analysis, the “worst aspects” and “suggestions for improvement” were combined together and called “quality improvement observations”. This enabled dichotomous analysis of multisource feedback. To compare the different versions of the *Perinatal Loss Workshop*, all observations were counted and analysed as dichotomous categorical variables with the proportion of “best aspects” expressed as a percentage of the total feedback (“best aspects” and “quality improvement observations”) to give a “total quality score”. Chi-square statistic for trend in proportions was analysed at a significance of 0.05.

### Acceptability

An anonymous participant satisfaction questionnaire used fixed response questions to rate the seven items of presentation technique, content, relevance, understanding, interactivity, tutor support and overall rating. A 5- point Likert-type scale rated the responses from 1 to 5 with 1 = “poor” and 5 = “excellent”. A composite mean score (SD) was calculated to measure acceptability of the workshop.

### Ethics approval

This project was exempt from Human Research Ethics Committee review according to the NHMRC National statement on research into humans [19] because the education was part of a clinical quality improvement program to implement national guidelines already endorsed in formal policy documents by all State and Territory health departments.

## Results

**Figure 2:**
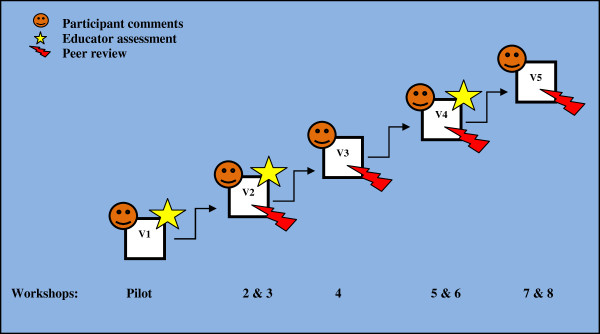
Multisource observation used to develop progressive workshop versions (V1 toV5).

From April 2008 to November 2010, 181 participants attended eight workshops delivered by 15 different tutors and held across five of the seven States and Territories in Australia. Five different iterative versions (Figure 2) resulted from our action research process.

### Version 1 (pilot workshop)

The pilot workshop was attended by 32 participants from the key stakeholder groups with representation from obstetrics, neonatology, midwifery, nursing, research, policy, education, support/ counselling and consumers.

### Versions 2 to 5

149 interprofessional participants included doctors (59.7%), midwives/nurses (28.2%), counsellors/social workers (10.1%) and researchers (2%). Version 2 is known as *IMPROVE* (Improving Perinatal Outcomes via Education). Versions 3 to 5 is called the *Perinatal Loss Workshop.*

### Standardised interactive education

Thematic analysis of multisource observations during the action research process identified eight recurring interactive education themes listed in Table 1. Each theme is coded as a quality domain (Table 1). Content standards are represented by two themes (1) Inconsistency within the educational framework (SCORPIO curriculum); (2) Content not aligning with PSANZ-CPG. The final content standards are summarised in Table 2. Teaching standards are represented by six themes listed in Table 1 being (3) Information overload; (4) Unacceptable station timing; (5) Didactic delivery; (6) Inadequate clinical relevance; (7) Poor teaching reproducibility; and (8) Incomplete engagement of participants. The workshop version at which each theme was resolved based on quality improvement observation is indicated in Table 1 and examples are provided to illustrate successful theme modification strategies. None of the themes were resolved after versions 1 or 2. Two teaching themes were resolved by version 3 and another by version 4. It was not until version 5 that both the content themes were resolved. The only partially resolved theme was the teaching theme, (8) Incomplete engagement of participants. The analysis of quality of the content and teaching resulting from the action research cycle is shown in Table 3. There is a significantly reducing trend in number of quality improvement observations and increasing trend of total quality scores from all three sources. By the final version 5, participants had no quality improvement observations whilst the educator and tutors did.

**Table 1:** Interactive education themes used to standardise content and teaching

**Standards**	**Interactive education theme**	**Quality domain**	**Resolved by version**	**Identification source**	**Examples of perinatal loss workshop theme modification**
**Content**	1. Inconsistency within the educational framework (SCORPIO curriculum)	Curriculum	5	Educator	• 3 stations using tell-show-do-feedback and 3 stations using problem-based learning
				Tutors	• Ensure a different participatory activity at each station
	2. Content not aligning with the clinical practice guideline	Alignment	5	Educator	• Omission from the workshop of an entire CPG Key Recommendation: “Institutional Perinatal Mortality Audit” (Table 1)
				Tutors	• Learning objectives providing incomplete coverage of CPG Key Recommendation
**Teaching**	3. Information overload	Overload	3	Educator	• Information limited to learning objectives
				Tutors	• Minimise slide numbers
	4. Unacceptable station timing	Timing	3	Participants	• Teaching station time extended from 20 to 30 minutes allowing completion of content delivery
				Educator	• Detailed written teaching plan with timing for the tutor to follow produced for every teaching station
				Tutors	
	5. Didactic delivery	Didacticism	4	Participants	• Information in slide presentation reduced and demonstrating using participant activity increased
				Educator	• 2 to 3 participatory activities in every station
				Tutors	
	6. Inadequate clinical relevance	Relevance	5	Participants	• Always use the context of an appropriate clinical scenario scenario
				Educator	• Difficult communication skill demonstrated by short DVD made using professional actors.
				Tutors	
	7. Poor teaching	Reproducibility	5	Tutors	• Detailed written teaching plan with information for the tutor to follow produced for every teaching station
					• DVD or structured role play scenarios and planned brainstorming activities reduces variation when using different tutors
	8. Incomplete engagement of participants	Engagement	-	Participants	• Every participant actively involved in at least 2 structured activities in every teaching station
				Educator	
				Tutor peers	

**Table 2:** Final content standard

**PSANZ-CPG key recommendations[**[11]**]**	**Teaching station**	**Teaching aim**	**Learning objectives**
**Perinatal post-mortem examination**	**1. Communicating with families regarding autopsy**	To provide information to enable parents to make an informed choice about perinatal autopsy	1. Know the relevant information to provide to parents to enable informed choice about perinatal autopsy.
			2. Understand the common barriers to obtaining consent for autopsy and be able to discuss solutions
			3. Apply the principles of compassionate communication in this setting
	**2. Autopsy and placental examination**	To describe care of the baby during and after autopsy and demonstrate the process of placental examination	1. Know the indications and the processes required for placental pathology
			2. Examine the placenta, cord and membranes systematically
			3. Explain the external appearance of a baby after autopsy
**Investigation of stillbirths**	**3. Investigation of perinatal death**	To explore the core investigations to be undertaken following a perinatal death	1. Understand the timing, type and reasons for the core investigations for stillbirth
			2. Explain the importance of amniocentesis
			3. Provide information about the role of non invasive investigations when autopsy is declined
**Investigation of neonatal deaths**	**4. Examination of babies who die in the perinatal period**	To demonstrate detailed clinical examination of babies including clinical photographs, measurements and investigations	1. Measure a baby and plot on Australian national birthweight percentiles
			2. Examine and use the recommended checklist for examination and investigation of perinatal deaths
			3. Know the recommended standardized clinical photographs
**Perinatal mortality classification**	**5. Institutional audit and perinatal mortality classification**	To provide an understanding of the purpose of institutional audit and how to use the PSANZ PNM classifications	1. Understand the value of classification of cause of death
**Institutional perinatal mortality audit**			2. Use the PSANZ classification for perinatal and neonatal death
			3. Know the perinatal mortality review process
**Psychological and social aspects of perinatal bereavement**	**6. Psychological and social aspects of perinatal bereavement**	To promote the need for support for psychological and social aspects of perinatal bereavement	1. Understand parental responses after experiencing perinatal death
			2. Know factors which contribute to the experience and outcomes of bereaved parents
			3. Appreciate support roles of different health professionals following perinatal loss

**Table 3:** Measurement of improving quality

**Workshop version**	**Observations**					
	**Participants**		**Educator**		**Tutors**	
	**No. of QI observations**	**Total quality score %**	**No. of QI observations**	**Total quality score %**	**No. of QI observations**	**Total quality score %**
**1**	26	45	18	49	*	*
**2**	45	59	15	53	33	34
**3**	12	63	*	*	35	41
**4**	3	92	3	91	11	76
**5**	0	100	*	*	2	91
**X**^**2**^	37.8^a^		15.6^b^		34.5^c^	
**p-value**	0.0001		0.0004		0.0001	

* Not done.

^a^X^2^ with 4df.

^b^X^2^ with 2df.

^c^X^2^ with 3df.

#### Participant observation

Participant observations were obtained after each workshop (Figure 2). There was a 55% increase in total quality scores (p <0.0001) from versions one to five (Table 3). Participants only ever made observations on four education themes (Table 1). The final version of the *Perinatal Loss Workshop* received exclusively positive comments from participants, including the two following statements: “*I liked the SCORPIO methodology- short, sharp, varied, interactive = power learning*” and “*a specialised area seen enough in practice to be necessary knowledge but rare enough so that clinical and communication skills and knowledge need workshop training.*”

#### Educator observation

An educator assessment was completed at the pilot and workshops two and five corresponding with versions one, two and four (Figure 2). There was a 42% increase in total quality scores (p=0.0004) from versions one to four (Table 3). All the interactive education themes were identified for modification except (7) Poor teaching reproducibility (Table 1). The final educator report after version four included the statement that “*the Perinatal Loss workshop was now professionally run by a well trained team and was conducive to effective learning*”.

#### Tutor peer observation

This process was initiated at the second workshop and repeated at workshops four, six and seven permitting observation of versions two to five (Figure 2). There was a 57% increase in total quality scores (p<0.0001) from versions two to five (Table 3). Tutor peer observation identified all eight interactive education themes (Table 1).

### Acceptability

The initial satisfaction questionnaire was completed in versions one to three by 83 (89.2%) participants. Results showed composite mean scores for both versions one and two were similar and indicated good to excellent acceptability with a combined score of 4.35 (SD 0.71) where five indicates an “excellent” rating. Composite mean score for version three was also good to excellent but was significantly better with a combined score of 4.81 (SD 0.43), p<0.0001.

From version four a revised and more detailed questionnaire with ten items was introduced (Additional file 1). A five-point Likert-type scale rated participant satisfaction with updating knowledge, updating clinical skills, updating communication skills, the relevance of the aims and objectives, coverage of the aims and objectives, the learning environment, the learning materials, the opportunity for interaction, the workshop set up and overall rating. It was completed by 79 (89.8%) of participants. The composite mean scores for both version four and five were similar and indicated good to excellent acceptability with a combined score of 4.66 (SD 0.53).

## Discussion

We have demonstrated that an interprofessional workshop can be developed for complex clinical guidelines. Participants found all versions of the workshop highly acceptable. The final versions which had the most complete coverage of the guideline content and which the interprofessional participants evaluated in the most detail, were universally deemed highly satisfactory to update knowledge, clinical skills and communication skills in an interactive learning environment. Following an action research approach, observations on the quality of the education were collated from participants, tutors and an independent educator and used to reflect and sequentially modify the workshop. Incremental resolution was demonstrated across eight quality domains which emerged during this cyclical collaborative process. These domains are: curriculum, alignment, overload, timing, didacticism, relevance, reproducibility and engagement which together provide a framework to standardise interactive teaching for complex clinical guidelines. They enabled maximal enhancement of the content and teaching pedagogy which is appropriate for the target audience. The improving quality of the workshop was demonstrated by the significantly reducing trend in number of quality improvement observations and increasing trend in percentage of positive observations from all three sources.

Grimshaw and Russell reported that educational strategies involving active professional participation, and that are closely linked to clinical decision making, are more likely to be associated with guideline implementation [20]. Research also indicates that involvement of the targeted professionals to test in practice is recommended before implementation, particularly for interprofessional guidelines [21]. Currently there is limited evidence on how to actually develop an educational strategy that fulfils these criteria. Action research is a method used to successfully blend academic and practitioner knowledge pursuit in healthcare as it is conducive to practitioner participation [22]. We integrated all this evidence to develop our interactive workshop and during the process an innovative approach evolved to standardise interactive education for complex clinical guidelines. In designing an effective education implementation strategy, the Cochrane systematic review on improving health outcomes from professional education meetings, acknowledges limitations in that many studies included have inadequate descriptions of their interventions making characterisation of the relevant factors such as level of interactivity and educational intensity not possible to analyse [23]. We feel that the detailed description of the educational intervention we have given illustrates the complexity and also the futility of attempting to assess individual components of an intervention. Ultimately a successful workshop is determined by a number of factors, as we have identified in our eight quality domains.

A major strength of this study was the multisource feedback which provided the observations for cyclical reflection and planning. Whilst more traditional participant observation is beneficial, it has limitations compared with tutor peer observation, in that our participants only recognised quality improvement issues relating to teaching and failed to acknowledge issues related to content. Tutor observation enables more comprehensive standardisation of interactive education as prior knowledge of the curriculum and the guidelines enable both content and teaching to be reviewed and modified appropriately. This is a critical point as effective transformation of guidelines into an education workshop will be dependent on the accurate representation of the content. Feedback obtained after continuing education workshops is usually only obtained from participants therefore significant inadvertent omissions in guideline content may persist unnoticed. A model of collaborative facilitation to reveal and address “blind spots” in learners’ knowledge has previously been reported in medical professional development using participative action research but this did not involve complex guideline implementation [24].

A further strength of this study was the interprofessional participant involvement. Doctors, midwives/nurses, counsellors/social workers, consumers and researchers all participated and through the action research process contributed collaboratively to the development of an interactive workshop that was consistently deemed relevant. Using this educational approach where different health cadres learn together and interact, we found role delineation can be discussed with the aim that participants left the workshop with a clear concept of the guideline areas relevant to their clinical role, but also with a greater appreciation of the roles of their colleagues and of consumer issues. Implementing complex guidelines requires collaborative care involving all members of the health care team and an understanding of the contributions of each provider is essential [25].

A limitation of this research is the relatively small sample size of each workshop. As each version was modified on the basis of only one or two workshops, it is possible that participants in different versions may have had differing levels of clinical and educational expertise which influenced their observation. Hence it is possible that our demonstrated improvement in quality for each version reflected differences in the participants themselves, rather than the educational method. Whilst this possibility is acknowledged, we believe it was unlikely this represented a significant source of confounding as there was consistent diversity in interprofessional background and experience of the participants. Also the improving quality of participant observations was replicated by the independent educator and the varying tutors involved in different versions.

There is conflicting evidence from the literature as to whether single or multifaceted interventions are more effective for implementing guidelines. This may influence policymakers’ decisions as to how they use this workshop given limited health resources [7,26-28]. Franke et al. concludes that in general, multiple strategies are more effective than single as they target different barriers to change [7]. In an obstetric setting this is supported by the systematic review of Chaillet et al. who would recommend that audit and feedback be implemented in addition to quality interactive education [28]. The Perinatal Loss Workshop quality interactive education is intended for national roll-out with the aim of implementing national guidelines to improve clinical practice. This workshop is fortunate to have the endorsement of the major clinical bodies in Australia responsible for the care of families who suffer perinatal loss (ANZSA, RANZCOG, SIDS and Kids, Australian College of Midwives). However, high-level advocacy from State and Territory Health Departments will be essential for widespread implementation to provide gravitas and resources for evaluation and reinforcing strategies.

## Conclusions

This description of the development of the *Perinatal Loss Workshop* has demonstrated how a complex clinical practice guideline (PSANZ-CPG) can be transformed into standardised interactive education based on the SCORPIO method. This has resulted in a workshop that is highly acceptable to an interprofessional audience throughout Australia. It is hoped that other professional groups can learn from this experience and recognise the importance of multisource participatory action research to optimise educational methods. The ultimate aim of clinical practice guidelines is to improve the care and outcomes that patients and their families experience. Thus it is essential that clinical behaviours and/or patient satisfaction are objectively evaluated following wide-scale implementation of an educational workshop and that the cyclical educational improvement process continues.

## Competing interests

The authors declare that they have no competing interests.

## Authors’ contributions

Concept and design (JIV, HEJ); collection of data (JIV, HEJ, JH, AG, SA); analysis and interpretation of data (JIV, HEJ, CRG); drafting of the manuscript (JIV); critical revision of the manuscript for important intellectual content (HEJ, CRG,AG, JH, DH); statistical analysis (JIV); provision of study materials (JIV, HEJ, JH, AG, SA); supervision (JIV; HEJ). All authors have read and approved the final manuscript.

## Pre-publication history

The pre-publication history for this paper can be accessed here:

http://www.biomedcentral.com/1472-6920/12/108/prepub

## Supplementary Material

Additional file 1 Final Evaluation. Click here for file
