# Supplementary material for: A method for developing standardised interactive education for complex clinical guidelines
Source: BMC Med Educ. 2012 Nov 6;12:108. doi: 10.1186/1472-6920-12-108 (PMC3533506; doi:10.1186/1472-6920-12-108)
Supplement: Additional file 1 — Final Evaluation. [file 1472-6920-12-108-S1.pdf]

Evaluation

	**Please rate the following aspects of this program:**	**Inadequate**	**Poor**	**Average**	**Good**	**Excellent**
1.	This workshop has updated my <u>knowledge</u> in the management of perinatal loss.	☐	☐	☐	☐	☐
2.	This workshop has assisted me with updating my <u>clinical skills</u> in managing perinatal loss	☐	☐	☐	☐	☐
3.	This workshop has assisted me with updating my <u>communication skills</u> in managing perinatal loss	☐	☐	☐	☐	☐
4.	I found the aims and objectives of the workshop to be:	☐	☐	☐	☐	☐
5.	The workshop <u>coverage</u> of the aims and objectives was:	☐	☐	☐	☐	☐
6.	The learning environment created by the tutors was:	☐	☐	☐	☐	☐
7.	The opportunity for hands on or interaction was:	☐	☐	☐	☐	☐
8.	The learning materials for the workshop were:	☐	☐	☐	☐	☐
9.	The venue and workshop set up was:	☐	☐	☐	☐	☐
10.	Overall I would rate the workshop as:	☐	☐	☐	☐	☐

Please describe the key message you learnt at each of the workshop components.

	**Perinatal Loss Workshop**	**Key Message**
11.	Introduction	
12.	St 1: Communicating with families about autopsy	
13.	St 2: Autopsy and placental examination	
14.	St 3: Investigation of perinatal death	
15.	St 4: Examination of babies who die in the perinatal period	
16.	St 5: Institutional audit & PNM classification	
17.	St 6: Psychological and social aspects of perinatal bereavement	
18.	Formative: group oral	

Please describe any aspect of the workshop that you think could be improved.

Perinatal Loss Workshop	Could be improved
19. Introduction	
20. St 1: Communicating with families about autopsy	
21. St 2: Autopsy and placental examination	
22. St 3: Investigation of perinatal death	
23. St 4: Examination of babies who die in the perinatal period	
24. St 5: Institutional audit & PNM classification	
25. St 6: Psychological and social aspects of perinatal bereavement	
26. Formative: group oral	

27. How likely is it that you would recommend this workshop program to a colleague

Not Likely Highly Likely

1 2 3 4 5 6 7 8 9 10

28. **What was the most significant factor that influenced your rating?**______________________

____

____

____

Thank you for taking the time to complete this evaluation.
